# Behavioural determinants of the use of pain-reducing interventions—a survey among professionals who vaccinate children

**DOI:** 10.1007/s00431-025-06740-2

**Published:** 2026-01-13

**Authors:** Bianca van Vreeswijk, Sijmen A. Reijneveld, Netty Bos-Veneman

**Affiliations:** 1 Department of Health Sciences, University Medical Center Groningen, University of Groningen, Groningen, The Netherlands; 2 GGD Groningen, Groningen, The Netherlands

**Keywords:** Pain-reducing interventions, Childhood vaccinations, Preventive Child Healthcare professionals, Behaviour, Survey

## Abstract

Childhood vaccination is a very effective public health intervention. Fear of pain during vaccination reduces vaccine willingness and can be addressed by interventions. We aimed to identify behavioural determinants of the use of pain-reducing interventions by Preventive Child Healthcare (PCH) physicians and nurses and the associations of sociodemographic characteristics with these behavioural determinants. We invited all PCH professionals of one municipal health service (Groningen, the Netherlands; *n* = 180) to fill in an online questionnaire on behavioural determinants of the use of pain-reducing interventions, based on the ASE (Attitude–Social influence–self-Efficacy) model of behaviour. We evaluated the associations of their background characteristics with their responses using logistic regression analyses. Of the 83 PCH professionals, 95% considered it important to reduce pain during vaccination, 90% intended to liaise with children and parents about pain mitigation, and 85% reported a high self-efficacy regarding the use of pain-reducing interventions. Lack of time and knowledge about pain reduction, and difficulties in the use of pain mitigation were negatively associated with the use of pain-reducing interventions. Nurses were more likely than physicians to liaise with children and parents about pain mitigation during vaccination (odds ratio, 95% confidence interval 8.86, 1.62 to 48.4) and believe they are competent to mitigate pain during vaccination (6.27, 1.65 to 23.9).

*Conclusion*: Most PCH professionals acknowledge the importance of reducing pain during vaccination but experience various barriers in the use of pain-reducing interventions. Education of professionals might contribute to the adherence to guidelines regarding pain reduction.

**Table:** 

**What is Known:** • *Childhood vaccination is a very effective public health intervention.* • *Fear of pain during vaccination reduces vaccine willingness and can be addressed by interventions.*
**What is New:** • *Most Preventive Child Healthcare physicians and nurses acknowledge the importance of reducing pain during vaccination but experience various barriers in the use of pain-reducing interventions.* • *Education of professionals might contribute to the adherence to guidelines regarding pain reduction.*

**What is Known:**

• *Childhood vaccination is a very effective public health intervention.*

• *Fear of pain during vaccination reduces vaccine willingness and can be addressed by interventions.*

**What is New:**

• *Most Preventive Child Healthcare physicians and nurses acknowledge the importance of reducing pain during vaccination but experience various barriers in the use of pain-reducing interventions.*

• *Education of professionals might contribute to the adherence to guidelines regarding pain reduction.*

## Introduction

Childhood vaccination is a very effective public health intervention. A high vaccination rate is essential to protect people from infections and prevent outbreaks. Vaccination rates are decreasing globally and in the Netherlands [1–3]. The World Health Organization (WHO) has identified vaccination hesitancy as one of the largest threats to global health [4]. In turn, fear of pain is the primary reason for vaccination hesitancy in 10% of Dutch teenagers [5]. Similarly, fear of pain causes 8% of Canadian parents to refuse vaccination [6]. Thus, professionals who address vaccination pain in children may help to increase vaccination willingness [2, 5]. Relatedly, the WHO advises all National Immunisation Programmes (NIPs) to include pain-reducing interventions for all age groups [7], such as providing breastfeeding or formula feeding before, during, and after vaccination, vaccinating the child on the caregiver’s lap, and using distraction techniques [8].

Vaccination guidelines of the Centers for Disease Control and Prevention (CDC), the European Centre for Disease Prevention and Control (ECDC), and the Dutch National Institute for Public Health and the Environment *(RIVM)* advise using evidence-based, simple, and effective interventions that reduce pain during vaccination, but these interventions are used only to a limited degree [9–12]. A Spanish cross-sectional study among 89 nurses showed that professionals are familiar with the techniques to reduce pain during vaccination but do not use them [13]. Several quantitative studies conducted in the USA among paediatric care providers suggest that multiple barriers exist regarding implementing pain reduction strategies during vaccination [14, 15]. Childhood vaccinations in the Netherlands take place in a different setting. In the Netherlands, childhood vaccinations are provided free of charge and administered at the well-child clinic by Preventive Child Healthcare (PCH) physicians and nurses. No research has yet been conducted on barriers in this context. Besides, various factors on societal, organisational, and individual levels contribute to this stagnating implementation. On the individual level, behavioural factors are of main importance.

**Fig. 1:**
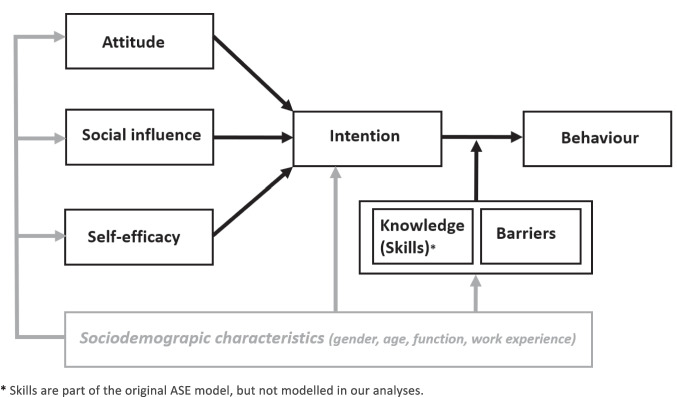
The ASE model as used to explain behaviour (in this study: use of pain-reducing interventions) and the position of sociodemographic characteristics (in grey) in this model as modelled in our analyses

The degree to which professionals adhere to guidelines can be studied using the ASE (Attitude–Social influence–self Efficacy) behavioural model, as described by Schellart et al. [16]. This model postulates that an individual’s behaviour is best predicted by their intentions (see Fig. 1) [17]. This study aims to identify behavioural determinants of and intentions to use pain-reducing interventions by PCH physicians and nurses, responsible for childhood vaccinations in the Netherlands, and the associations of sociodemographic characteristics with these behavioural determinants and intention to use.

## Methods

### Sample

In May 2021, we approached all 60 PCH physicians and 120 PCH nurses of the municipal health service serving the province of Groningen, the Netherlands. Professionals were approached by the human resources department of the GGD. In the Netherlands, vaccination practices vary between well-child clinics. During this study at GGD Groningen, approximately half of the vaccines were administered by PCH physicians and the other half by PCH nurses.

This study was conducted in accordance with the research code of the University Medical Center Groningen.

### Procedure and measures

Data were collected using an online questionnaire on demographic characteristics and on behavioural determinants of and intentions to use pain-reducing interventions as described by the ASE model, with the exception of the determinant ‘skills’. Professionals were initially contacted by the human resources department of the GGD and received information about the study via a digital information letter. One week later, they received a link to the informed consent form. After giving consent, participants received the digital questionnaire. If necessary, a reminder was sent 2 weeks later. We asked ten questions about intention, attitude, social influence, self-efficacy, knowledge, and barriers (see Table 2). We derived the questions from a previously developed questionnaire that measured the determinants of smoking behaviour according to the ASE model [18], and adapted this where necessary. We piloted the modified questionnaire in a sample of PCH professionals and made adjustments where needed. Answer categories ranged from’totally agree’ to ‘totally disagree’ using a 5-point Likert scale. Scores were dichotomised to the categories ‘agree’ and ‘not agree’, with ‘neutral’ categorised as ‘not agree’. Background characteristics included gender, age in years, function (i.e., PCH physician or nurse), and work experience in years. Age and work experience were dichotomised by median split.

### Statistical analysis

We first described the sociodemographic characteristics of the respondents. Second, we evaluated the responses regarding the various behavioural determinants of and intentions to use pain-reducing interventions, as dependent variables. Third, we assessed possible associations of these with background characteristics of the respondents, as independent variables, using logistic regression analyses. We treated the questions regarding the determinant social influence both as one factor (Cronbach’s alpha 0.602) and as separate items. We reported every other item separately because of the low internal consistency in subscales (Cronbach’s alpha < 0.6). We considered *p* < 0.05 as significant. We used IBM SPSS Statistics 28.

## Results

### Sample

The online questionnaire was completed by 83 professionals, a response rate of 46%. All professionals reported administering vaccinations to children aged 0 to 18 years in the year prior to the start of this study. Table 1 shows their demographic characteristics. Most of them were female (96%), and on average, they had about 11 years of work experience. Age and work experience of the PCH professionals who completed the questionnaire corresponded with those of the entire study population, according to information from the human resources department. The mean age and work experience of PCH nurses who completed the questionnaire were 47 years and 11 years, respectively, compared with 45 years and 12 years, respectively, in the entire study population. For PCH physicians, the mean age and work experience were 42 years and 8 years, respectively, in both the group that completed the questionnaire and the entire study population.

**Table 1:** Characteristics of respondents

	Preventive Child Healthcare professionals total (*n* = 83)	Preventive Child Healthcare physicians (*n* = 26)	Preventive Child Healthcare nurses (*n* = 57)
Female *%*	96.4	92.3	98.2
Age *Median (SD)*	46 (12.3)	42 (12.7)	47 (12.2)
Work experience *Median (SD)*	10.5 (10.7)	8 (10.8)	11 (10.7)

### Behavioural determinants

Table 2 summarises responses with regard to the various determinants of behaviour. Of the 83 professionals, 79 (95%) considered it important to reduce pain during vaccination. Moreover, 54 of the 56 nurses (96%) and 19 of the 25 physicians (76%) report that they intend to liaise with children and parents about pain mitigation during vaccination. The child’s opinion contributes to the intention to use pain-reducing interventions for 75 of the 83 professionals (90%), and the opinion of the parents for 67 of the 83 professionals (81%). Although 69 of the 81 professionals (85%) report a high self-efficacy in mitigating pain, they also report important barriers: 38 of the 81 professionals (47%) claim to have too little time to mitigate pain and 45 of the 81 professionals (56%) find it difficult to use pain-reducing interventions. Of the 83 professionals, 24 (28.9%) report having insufficient knowledge about it.

**Table 2:** Comparison of behavioural determinants of the use of pain-reducing interventions by Preventive Child Healthcare physicians and nurses with different lengths of work experience

		Function			Work experience		
	Total^a^ (*n* = 83)	Physicians^b^ (*n* = 26)	Nurses^b^ (*n* = 57)	*p*	≤ 11 years^c^ (*n* = 42)^*^	≥ 12 years^c^ (*n* = 40)^*^	*p*
Intention *I have the intention to liaise with child and parents about pain reduction during vaccination (% agree)*	90.1	76.0	96.4	0.012^**^	92.7	87.5	0.385
Attitude *It is important to reduce pain during vaccination (% agree)*	95.2	84.6	100	0.997	97.6	92.5	0.262
Social influence *(% agree) (treated as one factor)* *The child’s opinion on how to cope with pain reduction during vaccination is important (% agree)* *The parents’ opinion on how to cope with pain reduction during vaccination is important (% agree)* *My colleagues’ opinion on how to cope with pain reduction during vaccination is important (% agree)* *The opinion of my family and friends on how to cope with pain reduction during vaccination is important (% agree)*	51.8 90.4 80.7 32.5 13.3	46.2 92.3 80.8 38.5 7.7	54.4 89.5 80.7 29.8 15.8	0.378 0.724 0.934 0.583 0.163	57.1 90.5 83.3 33.3 14.3	45.0 90.0 77.5 30.0 10.0	0.267 0.945 0.506 0.750 0.539
Self-efficacy *I feel competent to reduce pain during vaccination (% agree)*	85.2	68.0	92.9	0.007^***^	80.5	90.0	0.231
Barriers *I have enough time to reduce pain during vaccination (% agree)* *It is easy to reduce pain during vaccination (% agree)*	53.1 44.4	48.0 40.0	55.4 46.4	0.544 0.597	51.2 41.5	55.0 47.5	0.741 0.591
Knowledge *I have enough knowledge of pain-reducing interventions described in the NIP guideline (% agree)*	71.1	76.9	68.4	0.667	61.9	80.0	0.074

*p*-values < 0.05 marked in bold

^*^Numbers do not add up to the total of 83 because of one missing value; ^**^odds ratio 8.86; 95% confidence interval 1.62–48.4; ^***^odds ratio 6.27; 95% confidence interval 1.65–23.9

^a^Percentage of the total group of Preventive Child Healthcare professionals who agree with the question

^b^Percentage of the specific Preventive Child Healthcare professionals who agree with the question

^c^Percentage of Preventive Child Healthcare professionals with more or less work experience who agree with the question

### Associations of behavioural determinants with sociodemographic characteristics

Table 2 shows the associations of the sociodemographic characteristics with the behavioural determinants of and intentions to use pain-reducing interventions. Regarding the type of function of the profession, we found, first, that more nurses intended to liaise with children and parents about pain mitigation during vaccination than physicians (odds ratio (OR) 8.86; 95% confidence interval (CI) 1.62–48.4) and, second, that more nurses believed themselves to be competent in mitigating pain during vaccination than physicians (OR 6.27; 95% CI 1.65–23.9). We observed no statistically significant associations with other sociodemographic characteristics.

## Discussion

This study shows that most PCH professionals consider it important to reduce pain during vaccination, intend to liaise with children and parents about pain mitigation, and report a high self-efficacy. Lack of time and knowledge about pain reduction, and difficulties with the use of interventions are important barriers in pain reduction. Nurses are more likely than physicians to liaise with children and parents about pain mitigation and to believe they are competent to mitigate pain during vaccination.

We found that professionals generally consider pain reduction to be important. They have a high self-efficacy in offering pain reduction. This differs from North American studies. A study performed in the USA shows that fewer, i.e. 58%, of the vaccinating professionals, acknowledge the importance of pain reduction and almost a quarter viewed pain as being ‘just part of the process’ [19]. Similarly, in a Canadian study, half the public health nurses perceived a high self-efficacy in the use of pain-reducing interventions [20]. Differences in training and in work setting, such as the presence of a local champion in Groningen, may offer explanations.

We found that major barriers for using pain-reducing interventions included lack of time and knowledge, and difficulties in using pain-reducing interventions, despite the professionals’ high level of experienced self-efficacy. Our findings are in line with a US study showing professionals to experience lack of time, education, and pain prevention tools as the most important barriers [19]. Other quantitative studies conducted in the USA among paediatric care providers also identified time and availability of tools as the main barriers in the implementation of pain reduction during vaccination [14, 15]. Training can have direct and indirect effects on these obstacles and can thus facilitate adherence to guidelines regarding pain reduction.

We further found that more nurses than physicians intend to liaise with children and parents about pain mitigation during vaccinations and believe to be competent to use pain-reducing interventions. To the best of our knowledge, no evidence on this difference between types of professionals exists. Potential explanations regard either differences between students when entering the profession or differences during their further professional training and work. Regarding this, an Italian study involving 502 students shows undergraduate nursing students to have a higher empathic engagement than other undergraduate health professional students. This suggests that an aptitude for empathy already exists at the beginning of the training of nursing students [21]. Evidence regarding this on medical students still lacks. Health professionals’ empathy skills and their enhancement deserve further study as a way to increase the use of pain mitigation during vaccination.

### Strengths and limitations

The validity of the collected data is strengthened by our use of a well-studied behavioural model. Another strength of the study is that it involved both PCH physicians and nurses.

Our study also has some limitations. First, the response rate of 46% is relatively low. This may have led to selection bias because professionals who consider pain reduction important were more likely to participate. Second, we assessed some concepts with only one question, which may have increased measurement error and thus reduced the likelihood of identifying behavioural determinants relating to pain mitigation. Third, our sample was of medium size, which may have limited the power of our study to detect weak associations. Finally, data were collected during the COVID-19 pandemic, which may have affected our findings. Professionals may have considered vaccinations more important during the pandemic. On the one hand, this may have led to a greater focus on pain reduction, but on the other hand, it may have led professionals to prioritize the vaccination itself over efforts to minimize pain.

### Implications

Our results have several implications. Our findings that most professionals consider pain reduction important, intend to liaise with children and parents regarding pain-reducing interventions, and believe they are competent to use these, show that professionals are very eager to reduce pain during childhood vaccinations. The stagnation of the implementation probably relates to the experienced barriers. With additional training, we can further enhance professionals’ knowledge and skills and also empathic engagement, and take away some of the barriers, as shown in previous implementation studies [22]. For example, a Canadian study showed that the percentages of public health nurses who feel confident about their use of pain-reducing interventions increased by 26% after training focused on pain management guidelines [20]. A review conducted in 2022 showed that education can improve nurses’ knowledge of, attitude towards, and practices in pain management in the short term, with its impact decreasing over time [23]. Thus, training has been shown to be effective, but continuous professional updates are needed.

Our findings also have implications for research. First, our findings on the importance of pain reduction, intention to liaise about pain reduction, and self-efficacy should be confirmed in other childhood vaccination settings. Second, it can be useful to explain professionals’ behaviour using other frameworks to assess factors that influence implementation outcomes. Other models provide a comprehensive framework of the multiple interacting components that influence behaviour, such as capability, opportunity, motivation, and contextual factors. This regards Capability, Opportunity, Motivation-Behaviour (COM-B) and Consolidated Framework for Implementation Research (CFIR) [24–26]. Third, we need longitudinal, observational research on the behaviour of vaccinating professionals to assess interventions they do and do not apply, before and after training.

## Conclusion

In conclusion, most PCH professionals acknowledge the importance of reducing pain during vaccination, intend to liaise with children and parents about pain reduction, and report a high self-efficacy. Nurses are more likely than physicians to liaise with children and parents about pain mitigation and to believe they are competent to mitigate pain during vaccination. Lack of time and knowledge about pain reduction and difficulties with the use of interventions are important barriers to pain reduction. Additional training focused on the perceived barriers is needed to increase the use of pain-reducing interventions in order to increase vaccine willingness.

## Data Availability

Scientific data can be requested from the corresponding author.
